# Variation of polypharmacy in older primary care attenders occurs at prescriber level

**DOI:** 10.1186/s12877-018-0750-2

**Published:** 2018-02-23

**Authors:** Su Miin Ong, Yvonne Mei Fong Lim, Sheamini Sivasampu, Ee Ming Khoo

**Affiliations:** 1 Healthcare Statistics Unit, National Clinical Research Centre, 3rd floor, MMA House, 124, Jalan Pahang, 53000 Kuala Lumpur, Malaysia; 2 Department of Primary Care Medicine, Faculty of Medicine, University of Malaya, 50603 Kuala Lumpur, Malaysia

**Keywords:** Epidemiology, Medication, Multimorbidity, Multilevel modelling

## Abstract

**Background:**

Polypharmacy is particularly important in older persons as they are more likely to experience adverse events compared to the rest of the population. Despite the relevance, there is a lack of studies on the possible association of patient, prescriber and practice characteristics with polypharmacy. Thus, the aim of this study was to determine the rate of polypharmacy among older persons attending public and private primary care clinics, and its association with patient, prescriber and practice characteristics.

**Methods:**

We used data from The National Medical Care Survey (NMCS), a national cross-sectional survey of patients’ visits to primary care clinics in Malaysia. A weighted total of 22,832 encounters of patients aged ≥65 years were analysed. Polypharmacy was defined as concomitant use of five medications and above. Multilevel logistic regression was performed to examine the association of polypharmacy with patient, prescriber and practice characteristics.

**Results:**

A total of 20.3% of the older primary care attenders experienced polypharmacy (26.7%% in public and 11.0% in private practice). The adjusted odds ratio (OR) of polypharmacy were 6.37 times greater in public practices. Polypharmacy was associated with patients of female gender (OR 1.49), primary education level (OR 1.61) and multimorbidity (OR 14.21). The variation in rate of polypharmacy was mainly found at prescriber level.

**Conclusion:**

Polypharmacy is common among older persons visiting primary care practices. Given the possible adverse outcomes, interventions to reduce the burden of polypharmacy are best to be directed at individual prescribers.

## Background

Polypharmacy can be defined in various ways and the more commonly used definition is the concomitant use of five or more medicines [1]. It is commonly associated with older persons [2], who are the largest per capita consumers of medicines [3], as they are more likely to have multimorbidity [4]. In the United States of America, although people aged 65 years and above constitutes 13% of the population, they contribute to about 34% of the use of prescribed medicines and 30% of over-the-counter preparations [5].

Polypharmacy in older person is associated with increased risk of drug-drug interactions, poorer adherence, increased risk of cognitive impairment, falls, poor outcomes [6, 7], and an increase in economic burden [8]. Older persons are at higher risk than the general population for these adverse events as age-related physiologic changes alter the pharmacokinetics and pharmacodynamics of drugs [9]. In addition, low health literacy, drugs misuse due to cognitive dysfunction in the older person also contribute to the increased risk of adverse events from polypharmacy [9].

Studies have shown that the probability of an older person developing an adverse drug reaction increases by 75% with polypharmacy, and up to 12% of all hospital admissions in older patients were attributed to adverse drug reactions [10, 11], of which half were avoidable [12]. Polypharmacy was also shown to be associated with higher all-cause mortality rate [13] and increased unplanned hospitalisation [14] in older person.

Nevertheless, polypharmacy may not necessarily reflect inappropriate practice. Older persons are likely to have multimorbidity that may necessitate the use of polypharmacy. Instead, by quantifying polypharmacy, we could provide a targeted scope for comprehensive medication reviews to identify patient at risk of potentially inappropriate medications [15, 16] and to perform systematic deprescribing [17].

The global population of people aged 65 years and above will constitute about 17% in 2050 with nearly two-thirds residing in Asia [18]. In Malaysia, it is estimated that 7% of the population will be aged 65 years and above by 2020 and by 2040 this number is likely to be doubled [19]. Thus, polypharmacy is expected to be a pressing issue for healthcare practitioners and policy makers. Healthcare in Malaysia is provided by a government-subsidised public sector where patients pay a minimal charge of US$0.30 per visit, and a private sector which operates through fees for services. Although these two sectors differ considerably in terms of structure and patients characteristics [20, 21], the challenges posed by polypharmacy affects both sectors equally.

Many studies have investigated the association between patient factors and polypharmacy [22–24] but few have examined the associations between prescriber and practice characteristics with polypharmacy. Polypharmacy may be influenced at patient, prescriber and practice levels. Therefore, determining the level at which variations in polypharmacy is greatest is important for targeting interventions. Two studies in Europe [25, 26] had studied the effect of higher levels on polypharmacy but such a study has not been done in a developing country. Hence this study aimed to determine the rate of polypharmacy among older primary care attenders in a developing country like Malaysia and examine its association with patient, prescriber and practice characteristics. We also intend to determine whether variation in polypharmacy is the greatest at the patient, prescriber or practice level.

## Methods

### Data source

This study used data from the National Medical Care Survey (NMCS) 2014 in Malaysia [20]. The NMCS was a nationwide cross-sectional survey of patients’ visits to primary care clinics. This survey used a multistage stratified random cluster sampling design, with practices used as the primary sampling unit. The sampled practices were randomly assigned a data collection day when all the encounters of the day would be recorded. Data collection was done using self-administered written standardised questionnaire. At the end of each consultation, prescribers filled the questionnaire with information on patient demographics, diagnoses and medications prescribed, information on individual providers and the facilities were also captured in a separate questionnaire. Further details about the method of the NMCS can be found at the Ministry of Health Malaysia, Clinical Research Centre website [20]. Process of care and medication coding were done using the International Classification of Primary Care – 2nd Edition Plus (ICPC-2 plus) and Anatomical Therapeutic Chemical (ATC) classification system respectively [27, 28]. Ethics approval was granted by the Medical Research and Ethics Committee of the Ministry of Health Malaysia (NMRR-09-842-4718).

The population of interest was patients aged 65 years and above, who presented to the primary care practices. Encounters that were not managed by doctors were excluded from the analysis. The primary outcome of interest was encounters with polypharmacy. We defined polypharmacy encounter as an encounter with prescription of five or more medications [1]. The type of medications commonly prescribed in these encounters was also analysed.

### Patient-level variables

Data collected were sociodemographic characteristics such as age, gender, ethnicity, education level, and presence of multimorbidity. Multimorbidity was defined as presence of two or more chronic conditions and these chronic diseases were identified based on ICPC-2 and ICPC-2 PLUS codes [29].

### Prescriber-level variables

Data collected included prescriber’s gender, average working hours per week, duration of practice in primary care denoted by experience, and place where first medical degree was obtained (Malaysia or abroad). The influence of qualification in family medicine specialisation was analysed only for the public sector as the numbers were negligible in the private practices.

### Practice-level variables

Data collected included practice workload, type of practice (group vs. solo), presence of a family medicine specialist (trained family physician with postgraduate degree in family medicine), practice working days per week (5, 6 or 7 days a week), and setting (rural or urban). Practice workload was determined by the total number of patient seen per day per full time doctors.

### Multilevel logistic regression models

Multilevel logistic regression analysis (MLRA) was used to estimate the odds of polypharmacy occurring in an encounter. It takes into account the hierarchical structure of the data, where patients are nested within prescribers, who in turn are nested within practices. The three-level model is applied to the public primary care practices. A two-level model of patients nested within practices was used for overall analysis and the private practices due to the high number of single-prescriber practices in the private practices. Using MLRA, we were able to quantify variation at each level within the hierarchical data and identify the level where greatest variation in polypharmacy occurs.

First, an empty model was built and subsequently three models were developed. Model 1 includes only patient variables, Model 2 includes patient and prescriber variables, and Model 3 (the full model) includes all three variables: patient, prescriber and practice. All variables were tested for multicollinearity, while higher levels’ residuals were also checked graphically for normality.

The odds ratios (OR) and 95% confidence intervals (95% CI) were calculated for fixed effects. We used the median odds ratio (MOR) to quantify the magnitude of the contextual effects for polypharmacy as it is considered a better measurement of variation in MLRA [30]. The proportional change in variance (PCV) between each model at prescriber and practice level were also determined. All estimation of variance for single sampling unit was scaled to give a conservative estimation.

Using the final model, residuals plot for each sector was drawn to visualize the variation. Public sector’s plot included variations between practices and between prescribers within practices. For private practices, only variation between practices was plotted.

The data were adjusted for complex survey design and analyses were done using STATA v14.0 (StataCorp LP, College Station, TX) [31] and R (version 3.3.0) [32].

## Results

### Descriptive analysis

There was a weighted total of 22,832 encounters of older persons seen by 3992 prescribers from 2914 practices. Table 1 summarises the characteristics of older persons, prescribers and practices. The median age of the study population was 71.2 years with a younger median age in public practices compared to private practices. More than half of the patients were females, but a higher proportion of patients in public practices had multimorbidity. The majority of the prescribers were female however more than three quarters of the prescribers in private practices were male. Prescribers in the private practices had longer primary care experience and clocked longer working hours per week than those in the public practices. Fewer doctors in the private had specialisation in family medicine. Private practices had lower workload per doctor but over 95% of these practices operated 6 to7 days a week. Private practices were predominantly solo practices and a majority was located in urban areas.

**Table 1:** Baseline characteristics at three levels: patients, prescribers and practices

Variables	Overall (%) (95% CI)	Public Sector (%) (95% CI)	Private Sector (%) (95% CI)
Patient Level	*n* = 22,832	*n* = 13,473	*n* = 9359
Polypharmacy	20.3 (17.0–24.0)	26.7 (22.9–31.0)	11.0 (7.1–16.6)
Age, median years (IQR)	71.2 (67.3–76.0)	70.5 (67.1–75.1)	73.0 (68.0–77.4)
Gender			
Male	46.4 (43.4–49.4)	46.0 (41.9–50.3)	46.9 (42.9–51.1)
Female	53.6 (50.6–56.6)	54.0 (49.7–58.1)	53.1 (48.9–57.1)
Ethnicity			
Malay	43.2 (37.0–49.7)	48.5 (40.0–57.2)	35.6 (27.0–45.1)
Chinese	43.0 (36.4–50.0)	35.3 (27.7–43.7)	54.2 (43.2–64.8)
Indian	9.6 (7.5–12.3)	12.5 (9.7–16.0)	5.5 (3.5–8.5)
Other	4.1 (2.6–6.4)	3.6 (1.8–7.2)	4.7 (2.6–8.4)
Education level			
No Formal Edu	25.5 (21.6–29.9)	24.8 (20.0–30.4)	26.5 (20.1–34.1)
Primary	42.5 (38.1–47.0)	47.9 (43.8–52.0)	34.6 (27.0–43.0)
Secondary	28.9 (22.9–35.8)	24.2 (20.1–28.9)	35.6 (23.4–50.0)
Tertiary	3.1 (2.3–4.3)	3.0 (2.0–4.5)	3.3 (2.0–5.5)
Multimorbidity*	47.4 (41.3–53.5)	62.9 (57.7–67.8)	25.0 (12.8–43.1)
Prescriber Level	*n* = 3992	*n* = 1590	*n* = 2401
Experience, median years (IQR)	14.0 (3.0–24.0)	2.0 (1.0–5.0)	22.0 (15.0–29.0)
Working hours, mean hours per week	48.1 (46.8–49.5)	43.1 (42.3–43.8)	51.5 (49.4–53.6)
Gender			
Male	41.9 (37.0–47.0)	30.8 (25.0–37.3)	76.1 (70.1–81.3)
Female	58.1 (53.0–63.0)	69.2 (62.7–75.0)	23.9 (18.7–29.9)
FMS	2.1 (1.3–3.4)	4.7 (2.9–7.5)	0.4 (0.1–2.7)
Place of graduation			
Foreign	50.3 (45.7–54.9)	51.3 (45.5–57.0)	49.6 (43.1–56.2)
Local	49.7 (45.1–54.3)	48.7 (43.0–54.5)	50.4 (43.8–56.9)
Practice Level	*n* = 2914	*n* = 564	*n* = 2350
Workload, median (IQR)	30.0 (20.0–43.0)	43.7 (28.0–54.6)	30.0 (18.0–40.0)
Sector			
Public	19.3 (17.4–21.4)	–	–
Private	80.7 (78.6–82.6)	–	–
Operation day per week			
Five	19.1 (16.3–22.3)	82.9 (73.0–89.7)	3.8 (2.0–7.4)
Six	47.2 (41.7–52.7)	5.5 (2.5–11.7)	57.2 (50.5–63.6)
Seven	33.7 (28.4–39.4)	11.6 (6.0–21.4)	39.0 (32.7–45.7)
Practice with FMS	11.0 (8.5–14.2)	47.1 (36.2–58.2)	2.4 (1.0–5.6)
Practice type			
Group	37.7 (32.7–42.9)	85.8 (77.1–91.6)	26.1 (20.6–32.5)
Solo	62.3 (57.1–67.3)	14.2 (8.4–22.9)	73.9 (67.5–79.4)
Urban location	81.8 (77.5–85.4)	56.8 (46.5–66.5)	87.8 (82.9–91.4)

CI*, confidence interval; IQR, interquartile range; FMS, family medicine specialist; Multimorbidity refers to having 2 or more chronic conditions*

Overall, 20.3% of the older persons presented in primary care have polypharmacy. The rate of polypharmacy in older persons was higher in public compared to private sector (26.7% vs 11.0%) with an adjusted OR of 6.37 (95% CI 1.17–34.71). However, the median of number of medications was three per patient for both sectors.

**Fig. 1:**
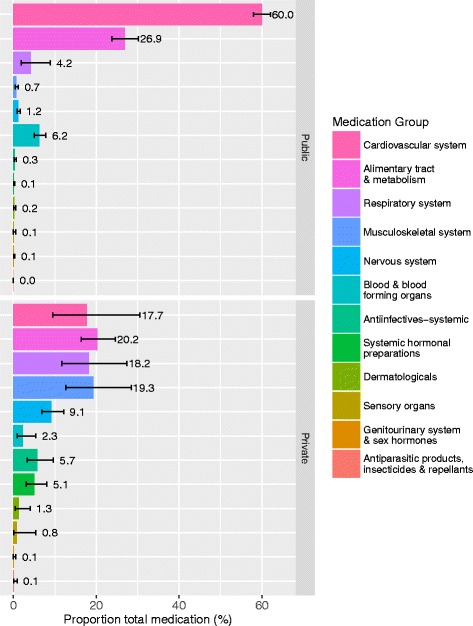
Types of medications used in patients with polypharmacy by sectors

The most used class of medications was from the cardiovascular system (50.3%). Figure 1 shows that in public sector almost 90% of the medications prescribed were for cardiovascular and alimentary tract and metabolism system; and the five most frequently prescribed medications in descending order were amlodipine, metformin, lovastatin, perindopril and hydrochlorothiazide (Table 2). In the private sector, almost 80% of medications were for cardiovascular, alimentary tract and metabolism, respiratory and musculoskeletal systems; while diclofenac, paracetamol, prednisolone, theophylline and calcium in combinations with vitamin D and/or other drugs were the five most frequently prescribed medications in this sector.

**Table 2:** Top 20 medications prescribed in public and private sector primary care practices

Medication Name	Proportions (%)	95% CI
Public Sector		
1. Amlodipine	10.5	(9.43–11.57)
2. Metformin	10.4	(8.94–12.04)
3. Lovastatin	10.0	(8.43–11.72)
4. Perindopril	7.9	(6.77–9.17)
5. Hydrochlorothiazide	6.5	(5.30–7.91)
6. Gliclazide	6.1	(4.97–7.43)
7. Acetylsalicylic Acid	5.4	(4.32–6.69)
8. Metoprolol	4.0	(3.07–5.24)
9. Simvastatin	3.5	(2.57–4.86)
10. Atenolol	2.6	(1.88–3.52)
11. Enalapril	2.4	(1.55–3.82)
12. Insulin (Human) - Intermediate Acting	2.0	(1.61–2.48)
13. Insulin (Human) - Intermediate- or Long-Acting Combine with Fast-Acting	1.7	(1.12–2.43)
14. Prazosin	1.6	(1.10–2.43)
15. Glibenclamide	1.6	(0.85–2.88)
16. Gemfibrozil	1.3	(0.46–3.47)
17. Furosemide	1.2	(0.80–1.70)
18. Nifedipine	1.1	(0.59–1.97)
19. Captopril	1.0	(0.55–1.74)
20. Potassium Chloride	1.0	(0.56–1.67)
Private Sector		
1. Diclofenac	6.0	(2.92–11.86)
2. Paracetamol	4.0	(1.99–7.96)
3. Prednisolone	3.1	(1.94–4.94)
4. Theophylline	2.6	(1.00–6.52)
5. Calcium, Combinations with Vitamin D and/or Other Drugs	2.3	(1.17–4.61)
6. Bromhexine	2.3	(0.77–6.60)
7. Atorvastatin	2.2	(0.85–5.57)
8. Metformin	2.2	(1.13–4.09)
9. Simvastatin	2.1	(0.81–5.37)
10. Loratadine	2.0	(0.59–6.54)
11. Amlodipine	2.0	(0.90–4.35)
12. Dexamethasone	1.6	(0.35–6.92)
13. Enzymes	1.5	(0.64–3.34)
14. Clopidogrel	1.4	(0.57–3.64)
15. Meloxicam	1.4	(0.74–2.73)
16. Tonics	1.4	(0.24–7.42)
17. Cefadroxil	1.4	(0.24–7.42)
18. Insulin Glargine	1.4	(0.48–3.76)
19. Atenolol	1.4	(0.62–2.93)
20. Vitamin B1 in Combination with Vitamin B6 and/or Vitamin B12	1.2	(0.51–3.03)

*CI, confidence interval*

### Multilevel regression analysis

#### Associations

Table 3 displays the results of the MLRA. Overall, higher odds of experiencing polypharmacy were associated with patients of female gender (OR 1.76), primary education level (OR 1.61) and multimorbidity (OR 14.21).

**Table 3:** Factors influencing rate of polypharmacy in older patients with multilevel adjusted final models

Variables	Overall, OR (95% CI)	Public sector, OR (95% CI)	Private sector, OR (95% CI)
Patient level			
Age, median (IQR)	0.99 (0.96–1.02)	1.00 (0.96–1.04)	0.99 (0.94–1.04)
Gender	Reference: Male		
Female	1.49 (1.11–1.99)*	1.76 (1.17–2.65)*	1.08 (0.53–2.19)
Ethnicity	Reference: Malay		
Chinese	0.94 (0.58–1.53)	0.93 (0.50–1.74)	1.65 (0.23–1.63)
Indian	1.11 (0.56–2.18)	1.21 (0.53–2.77)	0.45 (0.06–3.29)
Other	2.16 (0.49–9.52)	1.02 (0.35–3.02)	4.20 (0.62–28.24)
Education level	Reference: No formal education		
Primary	1.61 (1.12–2.32)*	1.68 (1.00–2.83)	1.69 (0.61–4.67)
Secondary	1.44 (0.93–2.23)	1.85 (0.99–3.46)	1.73 (0.59–5.05)
Tertiary	1.08 (0.47–2.5)	1.62 (0.51–5.17)	0.60 (0.07–5.34)
Multimorbidity	Reference: No		
Yes	14.21 (8.46–23.88)*	36.37 (17.39–76.09)*	5.54 (2.03–15.13)*
Prescriber level			
Experience	1.01 (0.97–1.05)	1.02 (0.96–1.09)	1.00 (0.96–1.06)
Working hours	1.00 (0.97–1.03)	0.97 (0.92–1.02)	1.01 (0.97–1.04)
Gender	Reference: Male		
Female	1.14 (0.63–2.04)	0.64 (0.32–1.29)	0.83 (0.27–2.55)
FMS	Reference: No		
Yes	2.81 (0.79–9.91)	4.66 (1.11–19.5)*	–
Place of graduation	Reference: Foreign		
Local	1.16 (0.76–1.78)	0.96 (0.52–1.80)	0.78 (0.31–1.97)
Practice level			
Workload	0.99 (0.97–1.00)	0.98 (0.97–1.00)	0.98 (0.96–1.01)
Sector	Reference: Private		
Public	6.37 (1.17–34.71)*	–	–
Operation day per week	Reference: Five		
Six	1.35 (0.50–3.62)	1.31 (0.76–2.24)	2.22 (0.17–29.28)
Seven	2.73 (0.88–8.45)	1.80 (0.20–16.27)	4.44 (0.29–67.57)
Practice with FMS	Reference: No		
Yes	1.56 (0.63–3.84)	0.93 (0.41–2.09)	7.68 (0.72–82.32)
Practice type	Reference: Group		
Solo	3.09 (0.94–10.11)	2.91 (0.16–53.1)	4.36 (0.98–19.31)
Urban location	Reference: No		
Yes	0.74 (0.33–1.64)	0.78 (0.44–1.41)	0.82 (0.19–3.50)

OR, odds ratio; CI, confidence interval; IQR, interquartile range; FMS, family medicine specialist; Multimorbidity refers to having 2 or more chronic conditions; * p-value < .05

In public sector, being female and having multimorbidity were associated with 1.8 and 36 times more odds to experience polypharmacy respectively. Prescribers with family medicine specialization had five-fold higher odds of polypharmacy prescribing while none of the factors at practice level were significantly associated with polypharmacy.

In private practices, the only factor that was associated with polypharmacy was patient with multimorbidity, where they had five times higher odds of having polypharmacy compared to patient with single morbidity.

### Variation

The variance of random intercepts at practice level remained at 3.30 (95% CI 1.77–6.15) after adjustment for all three levels variables. The odds of patients having polypharmacy increased about six times by changing from a clinic with a lower tendency for polypharmacy to a higher tendency one.

We further analysed the variance by each sector. Table 4 shows the analysis of variance of polypharmacy in public sector. A greater variation in the rate of polypharmacy was found between prescribers than between practices. The inclusion of patient, prescriber and practice variables into the models decreased the variance for polypharmacy between practices substantially as can be seen in the PCV. However, the variation for polypharmacy between prescribers was only explained to a small degree by patient level characteristics (12.8%) and not by prescriber or practice level variables. MOR in Model 3 shows that the odds of an older person experiencing polypharmacy increases by almost five times by randomly moving from a prescriber with lower propensity to one with higher propensity for polypharmacy within the same practice. Similarly, the odds of polypharmacy would increase by 1.5 times by randomly moving from a practice with lower propensity to one with higher propensity for polypharmacy.

**Table 4:** Variation between public practices and between its prescribers for each subsequent multilevel model

Random effects (intercepts)	Empty model (95% CrI)	Multilevel Model 1: (95% CrI)	Multilevel Model 2: (95% CrI)	Multilevel Model 3: (95% CrI)
Practice				
Variance	0.81 (0.13–4.95)	0.67 (0.12–3.67)	0.51 (0.05–4.82)	0.21 (0.001–43.59)
MOR	2.28 (1.4–7.67)	2.11 (1.37–5.78)	1.92 (1.24–7.46)	1.52 (1.03–422.31)
Prescriber				
Variance	3.13 (1.9–5.18)	2.73 (1.78–4.18)	2.70 (1.72–4.25)	2.75 (1.72–4.38)
MOR	5.06 (3.53–8.03)	4.54 (3.39–6.51)	4.51 (3.33–6.6)	4.56 (3.33–6.8)
Practice & prescriber				
Variance	3.94	3.39	3.21	2.96
MOR	6.16	5.40	5.16	4.83
Proportional change in variance (PCV) (%)				
Practice	–	17.3	23.9	58.8
Prescriber	–	12.8	1.1	−1.9
Practice & prescriber	–	14.0	5.3	7.8

*Note. MOR, median odds ratio; CrI, Credible interval*

The variance at practice level remained unchanged with the adjustments for patient and prescriber characteristics for private practices as shown in Table 5. It was only explained by a small degree when practice structural variables were included in Model 3 (PCV = 13.7%). The final model MOR shows that by visiting another practice with higher rate of polypharmacy, the odds of a patient experiencing polypharmacy were increased by almost eight times.

**Table 5:** Variation between private practices for each subsequent multilevel model

Random effects (intercepts)	Empty model (95% CrI)	Multilevel Model 1: (95% CrI)	Multilevel Model 2: (95% CrI)	Multilevel Model 3: (95% CrI)
Practice				
Variance	4.93 (1.96–12.43)	5.33 (2.13–13.35)	5.33 (2.13–13.34)	4.60 (2.04–10.38)
MOR	8.31 (3.8–28.86)	9.05 (4.02–32.63)	9.05 (4.03–32.58)	7.74 (3.91–21.63)
Proportional change in variance (PCV) (%)				
Practice	–	−8.1	0.0	13.7

*Note. MOR, median odds ratio; CrI, Credible interval*

**Fig. 2:**
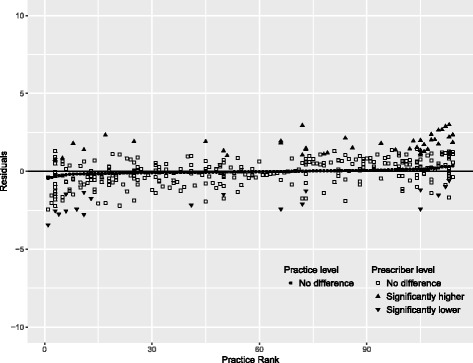
Variation in rate of polypharmacy between public practices and between prescribers within public practices

**Fig. 3:**
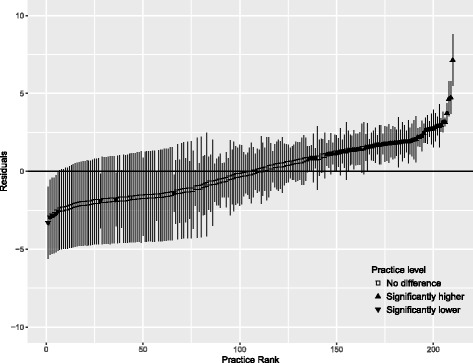
Variation in rate of polypharmacy between private practices. Error bars denote 95% confidence intervals for the residuals

Figure 2 shows the residuals plot for public sector which indicates that all the practices did not vary significantly from the overall average polypharmacy rate, but rather the variation mainly occurred at the prescriber level within each practice. Meanwhile, Fig. 3 shows a different picture for private practices, with 63 practices had significantly higher polypharmacy rates while nine practices showed significantly lower rates compared to the average in that sector.

## Discussion

One fifth of the older primary care attenders were found to have polypharmacy. This prevalence of polypharmacy is much lower than those reported in other nations in the Asia Pacific region where the rates were over 80% [23, 33]. This could be contributed by differences in health system infrastructure such as source of funding or universal health coverage status, morbidity and prescribing pattern, and differences in study designs and source of data. NMCS 2014 was a cross sectional study, while the other two studies with higher prevalence of polypharmacy were longitudinal studies. It is likely that the prevalence of polypharmacy in this study was underestimated because three other factors contributing to this burden were not taken into account. First, traditional and alternative therapies, which is often used in combination with western medicine in the Asian region were not taken into account [34]. Second, patients have freedom of choice of healthcare sectors including pharmacies for medicines due to dual healthcare system of public and private provision and a lack of primary care gatekeeping [35]. As a consequence, there was a lack of continuity of care and patients who doctor-hopped could have under-reported the number of medication or alternative therapies used during their encounters with health care providers. Lastly, medications for other chronic diseases not prescribed or repeated at current visit were not captured.

Polypharmacy was found to be more frequently encountered in the public primary care practices compared to the private practices. This could be due to difference in morbidity patterns, disease complexity and its severity [21] seen in these two sectors. Public health sector in Malaysia is highly subsidized and manage more chronic diseases compared to private practices where out-of-pocket payment is the predominant source of funding [36]. This led to the public sector handling more patients with multimorbidities and hence polypharmacy could occur and this might not be inappropriate [37]. While for patients visiting private practices, doctors might be influenced by cost factor when prescribing and could have reduced the number of medications prescribed. It has been found that patient’s request could affect prescriber decisions considerably regardless of the consequences [38].

Higher rate of polypharmacy was found in older female persons, which was also observed in another study [22]. This difference in prescription patterns can be attributed to gender-related health behavioural factors such as willingness to seek health care [22]. It is not surprising that multimorbidity increases the odds of polypharmacy in older patients because of the need to use multiple drugs to treat comorbid chronic conditions. In addition, prescriber who is a specialist in family medicine (FMS) or trained family physician had approximately five times greater odds of polypharmacy. This again is not unexpected because FMS in public sectors tended to see referrals and complex patient with multimorbidity, which might necessitate multiple medications regimen and increase number of medications prescribed. This is consistent with a study in in Taiwan that showed family physicians had a higher propensity to prescribe multi-drug treatments [33].

The greatest variation in the rates of polypharmacy was observed at prescriber level even after adjusting for prescriber and practice characteristics. The findings were similar to the two studies in Europe [25, 26] which looked into prescriber variations in prescribing patterns. This implies that the prescriber has a greater control over polypharmacy compared to the influence of the setting or institution in which the prescriber worked [25]. Hence, any interventions for polypharmacy need to target at prescriber level rather than practice level. The same applies to private sector as most of the private practices had a single prescriber; therefore, the variation observed for these practices is likely a reflection of the prescribing behaviour of the individual private prescribers. We were still unable to fully explain the reasons for this prescriber variability even though we accounted for more higher levels characteristics compared to the other two studies [25, 26]. Past studies had shown medical training and prescribers’ experience were associated with prescribing practices [39, 40]. However, this does not appear to be so in our study, aside from the family medicine specialist which we postulate is due to the characteristics of the patients they see. Further work in determining these factors is necessary, whether being informed with the latest medical knowledge, therapeutic inertia, prescribing pressure from patients or other reasons leading to prescriber variability in polypharmacy [25, 41].

In terms of the medications prescribed, it was not surprising that medications used for chronic diseases were most commonly prescribed in public sectors as chronic diseases such as hypertension, dyslipidaemia and diabetes were among the most frequently encountered diseases in public sector [20]. Studies had shown that older persons were highly susceptible to inappropriate prescribing of psychotropic substances [42, 43], but this was not observed in this study. It is known that overall use of psychotropic drugs is very low in Malaysia compared to other countries [44]. There are two possible reasons why low rates of psychotropic drug use were observed. First, mental illness is under-recognised from both patient and provider perspectives in the local setting [45]. Second, psychotropic agents including opioids and benzodiazepines are subject to strict control under the Dangerous Drugs Act and ongoing enforcement activities [44].

We found diclofenac was the most prescribed medication in older patients with polypharmacy in private practices. Non-steroidal anti-inflammatory drugs (NSAIDs) had been shown to be associated with increased risk of adverse events such as bleeding, myocardial infarction and increase in blood pressure, especially in the elderly [46]. This underlines the need to look into the appropriateness of prescribing in this study population. Currently, there are ongoing efforts to quantify inappropriate prescribing of medication in primary care practices in Malaysia, especially looking at the usage of NSAIDS. A study by Khoo et al. found that medication errors rates of up to 50% in Malaysian public clinics [47]. Therefore, further assessment on prescribing quality based on predefined criteria is necessary to determine areas for practice improvement.

Studies on drug use may be biased in settings with medication reimbursement policies [23]. In the present study, all medications provided within public health facilities were subsidised by the government. On the other hand, payment for medications in private practice is predominantly borne out of pocket by patients, followed by employer or third-party payers. In the latter, reimbursement is capped at a maximum cost rather than number of items. We acknowledge that whilst this may be a possible source of bias, we expect it to be minimal because neither number nor types of medications were restricted.

The strength of this study is the use of multilevel approach of practice, prescriber and patient, to examine polypharmacy in a developing nation. This allows us to target the appropriate level when developing intervention to gain maximum benefits. To date, there are few studies that describe prescribing variation at all three levels [25, 26], and these were conducted in developed countries. Another strength of this study is the use of a nationally representative primary care database, where data checks are in place for quality [20]. Medication data from NMCS 2014 were collected from prescribers’ prescriptions, which reduced recall bias and underreporting of medication compared to data collected from patient interview.

This study has its limitation. It is likely the study had underestimated the prevalence of polypharmacy in older primary care attenders because the use of over-the counter preparations, medications from other health facilities, herbal remedies, and traditional medicines were not accounted for. As the NMCS was a cross-sectional study, only the medications prescribed during the current visit were recorded. Information on patients’ full list of chronic medications was not available as patients were not required to be registered with a single primary care provider. Future studies looking at the full list of concurrent medications for chronic conditions are warranted. Electronic prescription records would enable longitudinal analysis of drug use in patients. However, this was not a feasible option for data capture because it is available in only about 60% of public practices and in half of the private practices, each using different proprietary systems [20]. With self-administered questionnaires, the limitation of selective reporting also could not be eliminated.

## Conclusion

Polypharmacy is common among older persons attending primary care and the greatest variation in its rates was observed at prescriber level. It was associated with multimorbidity in all practices, and in addition, being female gender and treated by trained family physicians in public sector. As polypharmacy may be appropriate in patients with multimorbidity, further studies should be undertaken to look into the appropriateness of multi-drug regimens. Nevertheless, regular review of polypharmacy at prescriber level is useful to identify patients at risk of inappropriate medications and possible associated adverse effects.

## Abbreviations

ATC: Anatomical Therapeutic Chemical Classification System
CI: Confidence Intervals
FMS: Family Medicine Specialist
ICPC-2 plus: International Classification Of Primary Care – 2nd Edition Plus
MLRA: Multilevel Logistic Regression Analysis
MOR: Median Odds Ratio
NMCS: National Medical Care Survey
NSAIDs: Non-Steroidal Anti-Inflammatory Drugs
OR: Odds Ratio
PCV: Proportional Change In Variance
